# Operationalizing mHealth to improve patient care: a qualitative implementation science evaluation of the WelTel texting intervention in Canada and Kenya

**DOI:** 10.1186/s12992-017-0311-z

**Published:** 2017-12-06

**Authors:** Kevin Louis Bardosh, Melanie Murray, Antony M. Khaemba, Kirsten Smillie, Richard Lester

**Affiliations:** 1 Department of Anthropology & Emerging Pathogens Institute, University of Florida, 2055 Mowry Road, Gainesville, FL 32610 USA; 2 Department of Medicine, Division of Infectious Diseases, University of British Columbia, E600B – 4500 Oak Street, Vancouver, BC Canada; 3 Women’s Health Research Institute, British Columbia Women’s Hospital, Vancouver, BC Canada; 4 Oak Tree Clinic, BC Women’s Hospital, Vancouver, BC Canada; 5 WelTel International mHealth Society, PO Box 50197 00100, Nairobi, Kenya

**Keywords:** Mobile health, mHealth, WelTel, Sms, HIV, Tb, Maternal and child health, ANC, Kenya, Canada

## Abstract

**Background:**

Mobile health (mHealth) applications have proliferated across the globe with much enthusiasm, although few have reached scale and shown public health impact. In this study, we explored how different contextual factors influenced the implementation, effectiveness and potential for scale-up of WelTel, an easy-to-use and evidence-based mHealth intervention. WelTel uses two-way SMS communication to improve patient adherence to medication and engagement in care, and has been developed and tested in Canada and Kenya.

**Methods:**

We used a comparative qualitative case study design, which drew on 32 key informant interviews, conducted in 2016, with stakeholders involved in six WelTel projects. Our research was guided by the Consolidated Framework for Implementation Research (CFIR), a meta-theoretical framework, and our analysis relied on a modified approach to grounded theory, which allowed us to compare findings across these projects.

**Results:**

We found that WelTel had positive influences on the “culture of care” at local clinics and hospitals in Canada and Kenya, many of which stretched beyond the immediate patient-client relationship to influence wider organizational systems. However, these were mediated by clinician norms and practices, the availability of local champion staff, the receptivity and capacity of local management, and the particular characteristics of the technology platform, including the ability for adaptation and co-design. We also found that scale-up was influenced by different forms of data and evidence, which played important roles in legitimization and partnership building. Even with robust research evidence, scale-up was viewed as a precarious and uncertain process, embedded within the wider politics and financing of Canadian and Kenyan health systems. Challenges included juggling different interests, determining appropriate financing pathways, maintaining network growth, and “packaging” the intervention for impact and relevance.

**Conclusions:**

Our comparative case study, of a unique transnational mobile health research network, revealed that moving from mHealth pilots to scale is a difficult, context-specific process that couples social and technological innovation. Fostering new organizational partnerships and ways of learning are paramount, as mHealth platforms straddle the world of research, industry and public health. Partnerships need to avoid the perils of the technological fix, and engage the structural barriers that mediate people’s health and access to services.

## Background

*Keep a watch also on the faults of the patients, which often make them lie about the taking of things prescribed. For through not taking disagreeable drinks, purgative or other, they sometimes die.*

– *Hippocrates, Decorum*

Since the days of Hippocrates, doctors have prescribed medicines that patients have just as quickly refused to take. Historical examples aside, non-adherence is a major global health problem, responsible for increased (and un-necessary) morbidity and mortality across a vast number of acute and chronic diseases around the globe [1]. An estimated 50% of patients today do not take medication as prescribed, costing health systems billions of dollars annually [1, 2].

Just as the outcomes of non-adherence are complex, so too are its causes; concentrating on the “lying” patient has all-too-often obscured this fact. HIV is an instructive example; over 90% of worldwide infections occur in resource-limited settings and an estimated 20% of patients are lost to follow-up within 12 months of initiating therapy [3]. A plethora of processes are involved and include (among others): socio-economic factors, health-care system characteristics, patient social networks, cultural models of health and disease, personal and psychological factors, clinic factors and drug regimen characteristics [4].

While the adherence challenge defies simplistic explanations, efforts to address it have largely focused on trying to change patient and healthcare provider behavior. Osterberg and Blaschke [2] divided these into four broad categories: patient education, dosing schedule changes, changes in staff practices and improved communication between clinicians and patients. Approaches range from: changes in care routines, more information, counseling, family therapy, self-monitoring, reminders and reinforcements [5]. Behavioral research on these interventions has highlighted multifaceted challenges associated with facilitating change; there is no “magic bullet.”

Within this context, the mobile phone technology revolution may offer a unique opportunity to build on these experiences through greater connectivity and data. This has generated significant interest from industry, government and public health practitioners. Mobile phone subscriptions have reached more than 6 billion, and 80% of new subscribers are from low or middle-income countries [6]. More people have access to a mobile phone than to adequate sanitation or clean running water.

The most common mobile health (mHealth) approach is a variety of daily and weekly one- or two-way SMS (Short Message Service) communication interventions that encourage patients to take their medication ([7, 8]). Other related innovations include digital technologies, such as electronic drug monitors, video observed therapy (VOT) and smartphone adherence apps.

MHealth has attracted much attention, but the field has been likened to the “Wild West.” Despite the ubiquity of cheap cell-phones, medical applications have yet to be widely adopted in the health policy domain or integrated into health systems beyond the realm of enthusiasm [9, 10]. Evaluations that go beyond the level of efficacy to explore issues of effectiveness and scale-up have also been few and far between ([11, 12]). As Tomlinson et al. [12] noted: “The current wave of mHealth interventions are the equivalent of black boxes. Each small entrepreneur or researcher includes whatever bells and whistles that their funding allows in an attempt to demonstrate efficacy.” Moving from efficacy to effectiveness trials, as the transition to scale process demands, requires considering how interventions, once developed, diffuse in the “real-world.” This includes understanding how social, cultural, technological and political factors and processes, operating at different levels, influence and mediate the “bells and whistles” that have been developed. In global health, this has become increasingly known as an “implementation science” approach [13, 14], and is also a central focus for medical anthropologists.

In this paper, we explore the implementation of a two-way SMS intervention (WelTel) in both the Global North (Canada) and Global South (Kenya). The WelTel model is one of the first examples demonstrating impact from mHealth on medication adherence to Antiretroviral Therapy (ART) and HIV viral suppression, through a randomized controlled trial in Kenya [15]. Since then, it has been expanded to include further work on HIV [16], tuberculosis [17], Maternal and Child Health [18], and Asthma [19]. This has included working with HIV-positive drug users in Canada [20] and remote pastoralists in northern Kenya. The *WelTel International mHealth Society* , a not-for-profit organization, was founded to scale-up the WelTel service in Africa ( http://www.weltel.org ).

**Fig. 1:**
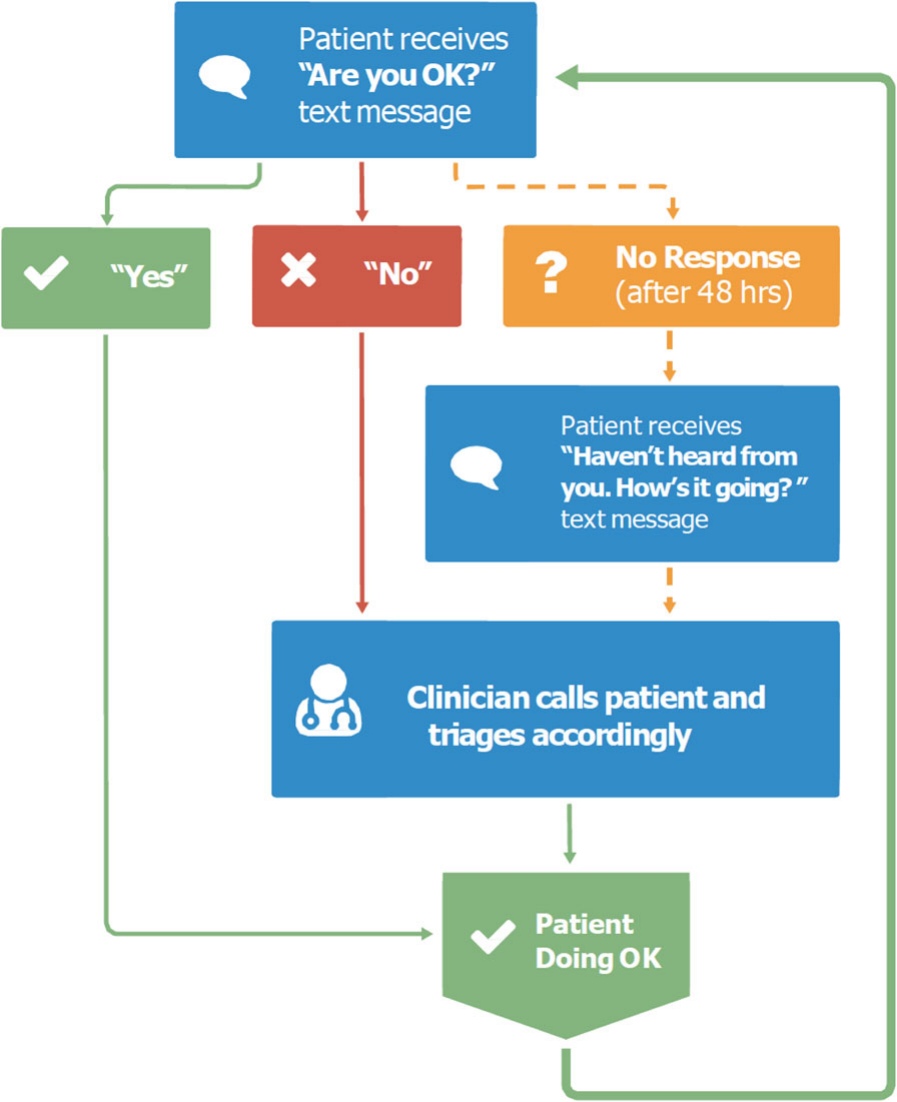
The core WelTel texting intervention for HIV/AIDS

The core of the technology is a simple “ask, don’t tell” approach to patient care developed through consultations, pilot testing and formative qualitative research [21, 22]. Patients receive a weekly text message that asks, “Are you okay?” (or “Mambo?” in Kiswahili). The message is sent every Monday and patients have 48 h to respond that they are either “well” (i.e. “ *Sawa* ” in Kiswahili) or if they have an issue or problem to discuss (i.e. “Shida” in Kiswahili). Another text is sent on Wednesday to remind clients that have not responded, and follow-up phone calls are initiated to contact the patient if no contact is made (see Fig. 1 in English). A key feature of the WelTel service is that it is easy-to-use and manage.

Here we report the results of a qualitative study exploring how the WelTel intervention was perceived, diffused, adopted and used by different health system actors in Canada and Kenya. Our aim was to provide a unique, comparative (Global North and South) perspective on the “real-world” complexity (or “messiness”) of implementation, and on the challenges and potential for scale-up, of an established mHealth application.

## Methods

### Project portfolio: The WelTel network

**Fig. 2:**
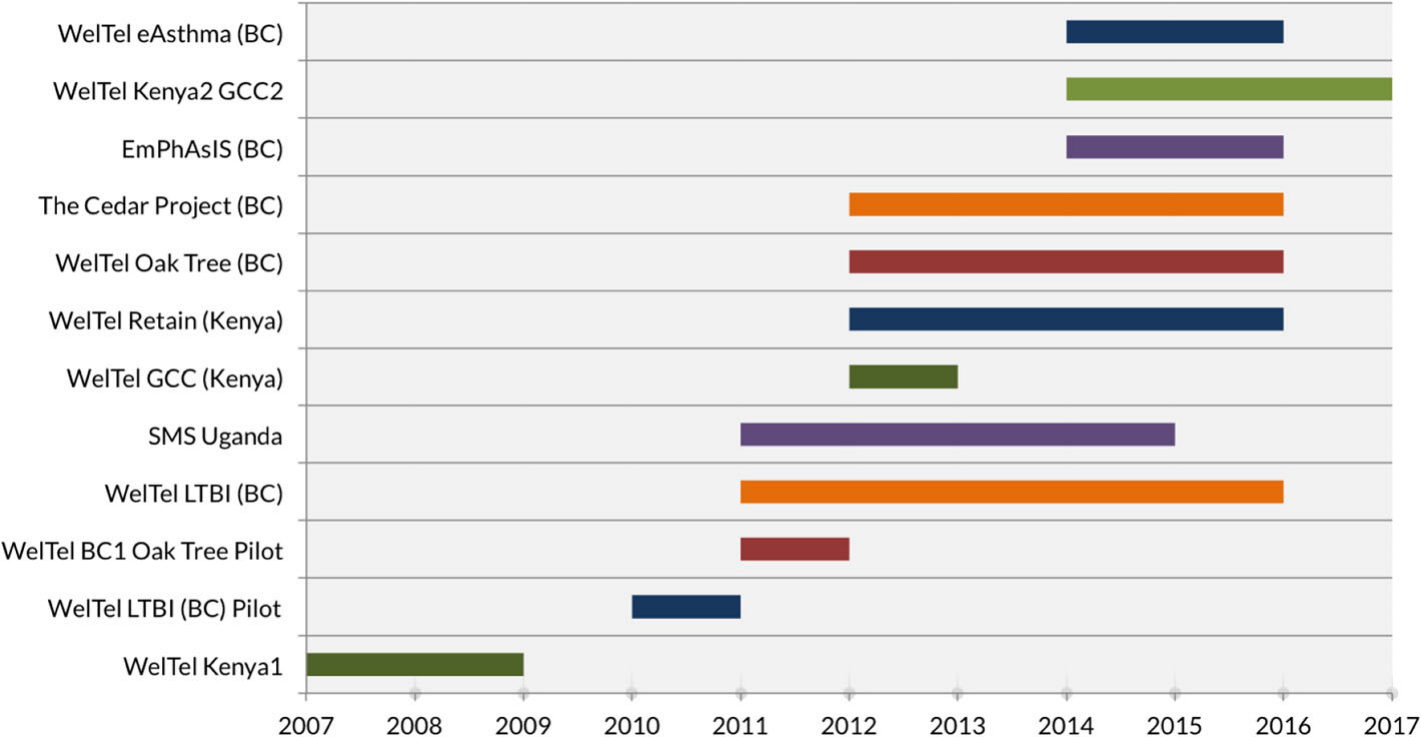
WelTel intervention projects

This study sought to comparatively explore enabling factors and challenges associated with implementation across a number of related but different projects. In total, we conducted interviews with stakeholders involved in six ongoing WelTel projects in Canada and Kenya: WelTel eAsthma, WelTel Kenya-2 Grand Challenges Canada (GCC), Cedar Project, WelTel Oak Tree, WelTel Retain and WelTel LTB1 (see Fig. 2 ).

The primary focus was on the most developed projects: WelTel in Kenya’s Northern Arid Lands (WelTel Kenya2 GCC) for HIV and Maternal, Neonatal and Child Health (MNCH), and to a lesser degree, on two HIV projects in British Columbia, Canada (Oak Tree and Cedar). The Kenyan project was primarily aimed at scaling-up and finding ways to integrate the service within the local health system, while Oak Tree and Cedar were still very much focused on generating evidence and proof-of-concept. We also explored the relationships between the Kenyan and Canadian projects. Projects are described throughout the text; here we provide a short summary:

1. *WelTel eAsthma* was a small-scale Randomized Controlled trial (RCT) with patients with severe Asthma in BC, Canada. The trial included weekly texts to patients and a web-based platform that provides access to patient action plans.
2. *WelTel Kenya2 Grand Challenges Canada* was a $2 million dollar “transition to scale” investment in partnership with Amref Health Africa/ Aphia plus Imarisha project, a major health sector NGO in Kenya funded by USAID. The project aimed to scale-up in the Northern Arid Lands (NALs), a remote pastoralist region with poor health indicators. At the time of research, WelTel was being used for nearly 700 HIV patients and some 1600 pregnant women in Isiolo District Hospital (IDH), a referral hospital in Isiolo County home to roughly 200,000 people. Activities were just beginning to expand to other sites.

3) *Cedar Project* built on existing HIV action research for indigenous people in BC, Canada [20]. The project has an ongoing cohort of 200 HIV and Hepatitis C vulnerable patients. The WelTel intervention was implemented to better connect young indigenous people who use drugs to Cedar case managers in a community-based setting. It included the weekly SMS texting service, and provision of free phones to vulnerable patients.

4) *WelTel Oak Tree* was a clinical effectiveness study exploring WelTel’s impact on clinical outcomes among vulnerable HIV-positive patients on ART. The project was run out of Oak Tree clinic at BC Women’s Hospital in Canada, and built on a successful pilot project [21, 22]. The study enrolled 85 HIV positive participants who were considered to be vulnerable and at risk for loss to engagement. It included the weekly SMS service, and provision of a free phone with text-message plan where required. In addition to clinical outcomes, this study collected detailed data on health care provider utilization and cost.
5) *WelTel Retain* was a National Institutes of Health (NIH) funded RCT at Kibera Community Health Centre in Nairobi, Kenya. The study explored whether the WelTel intervention improves retention in care of HIV-infected individuals ( *n* = 700) who have not yet started ARTs [16, 17].

6) *WelTel LTB1* was a two-year RCT study to investigate the impact of WelTel on Latent Tuberculosis Infection (LTBI) treatment completion rates. The project was implemented in collaboration with the BC-CDC and implemented at two clinics in Canada [16, 17].

### Conceptual framework

Research was informed by the Consolidated Framework for Implementation Research (CFIR), a meta-theoretical framework particularly well-suited to a comparative, cross-project evaluation. The CFIR includes five domains (intervention characteristics, outer setting, inner setting, characteristics of the individuals involved, and the process of implementation), which are divided into over 30 different constructs, or “sub-domains” (see [14]).

### Key informant interviews

Based on the CFIR, our study methodology involved a comparative case study design. To guide this, we developed three key informant interview guides, for health administers/managers, researchers and clinicians to be used across the various projects. These were divided into five sections: impressions before implementation, impressions during the early stages of implementation, the intervention-health system interface, the functionality of the technology platform and scaling-up.

We conducted 32 key informant interviews in British Columbia, Canada (11), and in Isiolo and Nairobi, Kenya (21), between February and April 2016 (see Table 1 ). We purposively selected our informants to cover a range of perspectives. Interviews lasted between 45 min to one-and-a-half hours. All interviews were conducted in private, and data collection included manual notes. Consent forms were signed for formal interviews, although we supplemented these with a more ethnographic approach, generating data through casual conversations at Isiolo District Hospital (IDH), with other stakeholders and with the WelTel team in Canada.

**Table 1:** Qualitative Interviewees by Category and Country

Key Informants	Canada	Kenya	Total
Researcher	9	1	10
Weltel staff		8	8
Clinic staff	2	10	12
Clinic manager		1	1
Government official		1	1
	11	21	32

Semi-structured interviews were done with nine researchers involved in current projects. In Canada, interviews also included two clinic staff responsible for managing the platform. A total of eight WelTel staff in Kenya were interviewed. Research at Isiolo District Hospital (IDH) included 10 different staff members at the Antenatal clinic (ANC) and HIV clinic. We also interviewed health managers and government officials. The focus on researchers in Canada and WelTel staff and clinic staff in Kenya (Table 1 ) reflected different levels of knowledge engagement. In Canada, most of the researchers we interviewed were intimately involved in the implementation of the pilot projects, whereas in Kenya this was the responsibility of WelTel staff working with the local clinics.

### Data analysis

We used a modified approach to grounded theory for data analysis. This involved open coding, preformed manually on data collection notes by a trained qualitative researcher (KB), in order to generate a key list of codes. A field-note diary was also kept, for brainstorming and reflection. This included case-based and analytical memos. This process facilitated the exploration of relationships and connections between different themes and subthemes, generating our analytical interpretations. Importantly, analysis was validated through a follow-up workshop in Kenya with IDH and WelTel project staff in July 2016, and through providing drafts of this article to a sub-group of key informants in Canada, as a form of member checking.

### Ethical approval

The study was approved by the University of British Columbia’s Clinical Research Ethics Board (H16–00189), and Amref’s Ethics and Scientific Review Committee (AMREF-ESRC P161/2015).

## Results

Based on our qualitative data analysis, we divided this paper into five sections. First, we discuss the provider-patient relationship and how the Weltel intervention was perceived to influence this. Second, we explore how the intervention interacted with service provision and organization at the clinic level. Third, we go on to discuss the socio-technical dimensions of WelTel and how the technology itself was perceived and used. Fourth, we present data on the important role of evidence and data in generating support and legitimacy for the intervention from different stakeholders, and how this process influenced implementation. Lastly, we turn to the issue of scaling-up and discuss the important role of networks, policy and politics.

### Improving the “culture of care”

Our interviews focused heavily on how health sector actors perceived the utility and benefits of using WelTel. We found that these extended far beyond the immediate SMS communication to influence the “culture of care” at local clinics, including services, standards, organization, management and accountability. In line with previous studies [22], the intervention was widely perceived as a “tool” that empowered patients by better connecting them to medical staff between appointments and increasing their access to medical expertise and outreach. In both Canada and Kenya, SMS is widely used, making it easy to connect through text [23], although access was believed to be lower in Kenya among pastoralist communities and women.

By allowing patients to seek immediate feedback on questions and problems, WelTel facilitated a *“sense that someone cares”,* and helped patients direct their own care. 1 This included addressing questions related to appointments, medical issues, medication side effects, and social issues. Importantly, our interviews also stressed the ways in which it facilitated *greater* access to care. In Kenya, one informant summed this up with the catchy saying: “the more you talk, the more friendly you become” ( *Kuongea ni Kuongeza Urafiki* , in Swahili). Similar euphemisms were used in Canada. Complex issues could be triaged on the phone, connecting patients to multiple healthcare providers, while also allowing patients with chronic conditions, like HIV, to build stronger rapport with their providers. In Kenya, and to a lesser degree in Canada, one of the most emphasized benefits was the ability for patients to circumvent long wait times for immediate health needs.

*“The flexibility [of the technology] is good. You can be texting a patient. They have a problem. You go and find that [specialist] in the clinic. You call and give the phone right to the health worker. They get immediate feedback! And if they need to be seen, they come right away.”*

(Interview, Clinician, Kenya)

Texting in Kenya was largely restricted to responding to the weekly check-ins, with occasional auxiliary texts to “encourage” patients to follow medical recommendations. In comparison, patient-clinic interactions were much more dynamic, common and personalized in Canada due to the explicit “patient-centric” approach of the clinics involved. In the Canadian HIV projects, patients were reported to regularly text about the *“joys and challenges of life”* (Interview, Clinician, Canada) while staff could also use the service to check-in with patients who were going through difficult periods, either clinically or emotionally.

Versatility of the SMS technology was described as a *“lifeline”* that allowed patients to engage on their own terms, when and how they needed assistance. They could be very active on the platform, and then remain silent until a future need arose. The benefits of SMS also allowed some more vulnerable patients in Canada to “open-up” about complex issues, due to the “emotional safety”, as one nursing staff described it, of the SMS technology. These benefits were widely discussed as a form of *“patient empowerment.”*

Improved bidirectional communication was viewed as improving the culture of care at clinics in multiple ways. This included impacts on staff motivation, performance, teamwork, job satisfaction, work routines and relations between staff and managers. Staff and management noted how the positive impact on patients facilitated an improved sense of work satisfaction, with positive secondary effects on performance. As one HIV counselor in Kenya mentioned:

*“It helps with our sense of teamwork and satisfaction of clients… Psychologically, you feel better connected to patients. And it really helps to motivate staff to ‘pull up their socks’ and care for the patients more!”*

In Canada, HIV staff discussed the improvement in workflow for dealing with complex patient issues, which previously required keeping track of multiple text-based and phone conversations. As an organizational tool, the intervention was noted as helping to save time for clinicians and outreach workers in Canada by systematizing patient communications:

*“We were texting before [with patients] but you could easily forget about someone’s [text]…[the] Weltel [computer-based platform] creates regularity. It’s an organizational tool that is much easier to use than scrolling down on my* [cell phone] *. The platform helps us never forget.”*

(Interview, Clinician, Canada)

In the resource-limited context of northern Kenya, noteworthy changes in patient care, with important secondary influences on hospital management, were emphasized. This included assisting with timely emergency medical outreach. One widely discussed example included how the texting service helped link the hospital to a remote pastoralist woman in childbirth, who was having life-threatening complications. An ambulance was mobilized and the woman delivered safely at the hospital and recovered. WelTel also helped justify financial investments for other forms of patient care. This included helping rejuvenate HIV psychosocial support groups, with support from an NGO-partner. Furthermore, the greater communication with HIV clients drove clinicians to better appreciate the full scope of HIV-TB co-infected patients at Isiolo District Hospital. This led to the purchasing an expensive GeneXpert machine to facilitate more prompt TB diagnosis at the hospital.

Perhaps most importantly from the patient perspective, the service allowed patients to report malpractices or administrative errors, facilitating changes in clinic guidelines and protocols. This is noteworthy, given that health worker performance is often low in many public hospitals in Africa and there are often few avenues for patients to provide feedback and complaints to management [24]. Phone communication, so Kenyan staff felt, could improve clinician accountability. One example included a clinical officer intern at IDH who prescribed the wrong HIV drugs to a patient; the patient contacted the hospital through the SMS service to flag the error. If not for the service, the error would have gone unnoticed (according to multiple interviews). In response, the hospital revisited its intern work-related policies.

Clinic staff also highlighted the ways in which the intervention raised awareness regarding individual patient circumstances. In Kenya, this included situations of extreme poverty, food insecurity issues and lack of access to drugs for opportunistic infections that had an impact on health outcomes. Kenyan staff questioned their ability to address these issues, and commented on the importance of clearly explaining the limitations of the intervention. As one staff member discussed:

*“How can you get [the patient] to appreciate the limitations [of WelTel]? For example, [the drug] dapsone is free but not always in stock…the patient will complain, “This person offered to respond to my [problem], but now they are not willing!” We tell them we will try our best, but we are not always able to address everything.”*

This social context also influenced enrollment and utilization patterns. Data from Isiolo District Hospital showed enrollment to be roughly 50% of the HIV patient population, despite the intention to enroll all patients who had access to a mobile phone. Staff noted a number of important disincentives for enrollment, related mostly to larger structural barriers to care (illiteracy, stigma and, for some remote pastoralist groups, lack of phone ownership). Despite the service being, for the most part, free, WelTel staff estimated that only 20% of HIV patients enrolled in the intervention immediately; 80% enrolled only after being encouraged by other patients.

Unlike in Kenya, HIV staff in Canada reported that most patients enrolled without any problem and that one of the major strengths was helping to *connect* patients to social services when they needed it. If they did not have food, they could be referred to a food bank or an emergency food grant, for instance. But in Kenya, where social services are few and far between and dependent on NGO grants, addressing these issues was more complex. With help from social workers and IDH, the WelTel intervention did provide some assistance to patients with healthcare costs through a waiver system for the most vulnerable and poor. But IDH clinicians recommended that future operations include greater linkage between the WelTel model of care and broader developmental goals to assist with broader social determinants of health. However such recommendations, in some ways, ran counter to a core objective of the intervention: to be easily implementable and scalable across large geographical areas and diverse health contexts, with minimal direct investment in human capacity.

Despite these differences, the ways in which patients benefited from the service in Canada and Kenya had similarities. In the original RCT trial in Kenya, *Shida* responses were recorded at 2% of all participants by 6-months [15]. Preliminary data showed a similar number of patients communicating with clinic staff on a weekly basis. One Canadian nurse stated:

*“At the beginning, some thought WelTel would be magic bullet for those that we had not connected with. It would be the missing piece, and for a handful, it was...For others, they didn’t use it. The funny thing is that you could not have predicted beforehand who would have benefited most! People use it very differently depending on the needs they are having at the time.”*

In this sense, the WelTel experience echoed broader findings on behavior change interventions in public health: impact was focused, incremental and highly variable depending on the needs and wants of particular end-users. Accurate figures on scaling-up and long-term public health impact would have to account for these nuanced utilization patterns, and how they change over-time.

### Organizing services

How the WelTel service improved the culture of care, and to what extent and with what effect, was influenced by the ways in which the service was organized at the clinic level. This mediated the “implementation climate” described by Damschroder et al. [14], including the “absorptive capacity for change and receptivity” of a health innovation.

The ways in which the service was delivered and integrated with the health system had significant implications for cost, quality of care and sustainability. In Canada, higher clinic capacity allowed the intervention to remain the prerogative of clinic staff. This allowed the Oak Tree HIV Clinic to continue WelTel with roughly 40 patients after research funding came to an end in 2015, as part of routine services. In contrast, researchers and staff in Kenya emphasized the challenges involved in integrating the service with clinic work routines. To address this, for example, the WelTel Retain Randomized Control Trial (RCT) in Nairobi paid a higher than average salary to study staff in the Kibera slum clinic to ensure the integrity of research protocol and quality results. But this type of incentive was not done at Isiolo District Hospital (IDH), where the main objective was to explore scaling up within existing systems.

Achieving this goal was, not surprisingly, difficult. Kenyan staff at IDH highlighted how a combination of human resource shortages, low morale, work culture norms and low technology skills meant that the original intention of the project (funded by Grand Challenges Canada) shifted; the *WelTel mHealth Society* needed to hire two local “expert patients” to enroll and triage patients. This is a common theme in global health projects, responsible for the large number of so-called “vertical” interventions [25].

We found that various geographic, cultural, social, economic and political factors of the Northern Arid Lands (NALs) were invoked in different ways to explain this. As a key informant in Kenya discussed it:

*“It’s about attitude – WelTel requires consistency and work discipline…This is very hard for [clinic] workers with ‘fluid schedules.’ You need someone with basic technology skills; someone educated about the benefits of technology and innovation. But this is just not prioritized in the staff mentality…the region is very remote.”*

At IDH more broadly, the running of both the ANC and HIV clinics depended heavily on one or two focal nurses who were overburdened with responsibilities. A manager summed this up:

*“At [the HIV clinic] if the in-charge nurse is not there, things will not work well. You have a situation where you depend on one person to run everything. We witnessed this very much – you find that when this person leaves, everything drops-off.”*

Another pervasive narrative was that staff lacked the technology skills to manage the service effectively without direct oversight support. Participants commented at length on the lack of “IT literacy” of hospital staff, especially in reference to another technology innovation introduced by Amref: IQ-CARE, an ambitious project to initiate a complete electronic records system to replace the current paper-based system. This initiative ran into multiple bottlenecks and was, for the most part, later abandoned.

While staff liked WelTel, there were a number who expressed reluctance to manage it if external support was withdrawn. Some explanations were not related at all to technology skills, or a widely discussed sense of being over-worked; rather, they were rather to social norms. One repeated reason was that nurses did not want to be “accused” of overusing airtime, or using the phone credit for personal use. In a resource-poor clinic, small material benefits were viewed as adding-up, with the potential to generate social conflicts [26]. All of this related to the internal legitimacy of the intervention, still considered an “external project” and not a core routine activity of IDH, overseen directly by management.

However by relying on WelTel staff, many IDH clinicians did not fully appreciate how WelTel worked at the time of research, despite it being implemented nearly a year prior. This contrasted with the Canadian HIV projects. Cedar, for example, had planned their own one-week systematic training for all staff members, which included inviting Oak Tree staff to attend (and provide mentorship), role playing to familiarize staff with the service, and group consensus on all triage protocols; two-weeks of piloting had then followed. While resource intensive, this enhanced training could be valuable in the Kenyan context.

In this sense, all projects emphasized the need for a “focal person” or “local champion” to manage and over-see the service, given the multitude of other administrative and work-related responsibilities. This appears be a major limiting constraint to the effective scalability of mHealth interventions. The differences in capacity of the ANC clinic at IDH in Kenya (over-crowded; long wait times; lower levels of care) and the HIV clinic (greater capacity and infrastructure; with direct donor support) required different responses. WelTel mHealth Society staff at IDH included an “expert patient” (HIV-positive individual) at the HIV clinic and a more educated Information Technology (IT) diploma holder at ANC, both of who were paid very modest (and different) monthly salaries. Responsibilities included giving health education talks, retrieving files, enrolling patients, managing the tablet platform, making follow-up calls and triaging patients. As found in other studies, challenges of motivation and capacity ensued [27]. These two paid volunteers were overseen and supported by WelTel mHealth Society staff in Isiolo (a project coordinator, IT/data analyst and administrator working from the Amref office in Isiolo). These individuals provided direct day-to-day management to ensure that activities went according to plan. They also planned scale-up, which began in mid-2016 in neighboring Marsabit and Samburu counties, just as research was being conducted. One major question for scaling to these sites revolved around whether or not to rely solely on existing clinic staff to implement WelTel or to pay auxiliary staff, which was felt to have major implications for cost, sustainability and effectiveness.

While the context in Canada was very different, clinic staff also emphasized a dependence on one focal person to ensure the service ran smoothly. The LTB1 study found that one administrator was instrumental in assisting with enrollment and follow-up. The Canadian HIV projects stressed the importance of having experienced outreach nurses play this role. At Cedar, patients endearingly called the main outreach worker managing the platform, the “phone lady.” Interviewees stressed the importance of linking “trust and relationships” with “the cold technology” in order to facilitate improved care. You needed a “human touch” to the intervention, as one informant termed it. While focal staff originally thought WelTel was going to overburden their normal activities, they found that once accustomed, it consumed modest amounts of time and streamlined existing forms of patient communication, as discussed above. One focal nurse in Canada estimated that she spent approximately 2 to 3 h per week managing the platform, calls and follow-ups.

### Tailoring technology

So far, we have discussed how the WelTel intervention, a relatively easy-to-use and evidence-based mHealth application, influenced quality of care and organizational cultures. Our data showed that both of these domains were influenced, in different ways, by the technical characteristics of the WelTel platform. This included issues of functionality, end-user friendliness, complexity and adaptability. A major question, echoed throughout our interviews, regarded the degree to which the intervention could be tailored and customized to specific groups of patients and in ways that met the wants and needs of clinic staff and management.

The experience of WelTel showed that considerations for scaling mHealth should be located in the challenges of effectively designing and refining the platform, or product, which can take much longer than sometimes appreciated. Technical adaptation dated back to the early Kenyan RCT trial (2005–2007) where nurses had to manually text patients weekly using paper registers. Multiple iterations of the software and platform were subsequently developed, with requests from each project and partnership to improve functionality. The most significant system innovation was the software redevelopment that shifted the WelTel platform from facility-based (WelTel V1.0) to a centralized-server system (WelTel V2.0). WelTel V1.0 had to be downloaded onto individual computers at each clinic. During the Kenyan CDC Foundation study (2011–2012) in seven clinics around Nairobi, the initial version had become difficult and expensive to maintain, having to be physically serviced and dependent on the city’s irregular power supply. Medical superintendents were reluctant to have patient data posted online since it would run against existing Ministry of Health policies. In response, the WelTel team designed a centralized-server system, housed on a private server at the Amref office in Nairobi. This allowed for security, consistent electricity and high-speed internet connection. Redevelopment also used new and more efficient programing language, allowing flexible upgrades.

WelTel mHealth society staff spoke about this period as a lesson in “back-scaling.” The initial intention of scaling-up from the CDC pilot had to be reconsidered, as substantial technical re-investment was needed. This informed the current “off-line” software platform at Isiolo District Hospital (IDH), which allows patients to be enrolled and followed-up on tablets without immediate access to the internet; enrolment is uploaded onto the server when connection is available. This was seen as making WelTel ideally suited for deployment in remote regions, like Northern Kenya, compared to many other similar systems being developed in Africa.

Interviews in Kenya also highlighted three other technology-related issues relevant to scale-up. First, there were different views regarding the ease to which the low-cost tablets piloted at IDH could be scaled-up. Some clinic staff emphasized that it would be better to utilize existing clinic phones (provided by NGOs like Amref), while others stressed that tablets were superior and training could easily be provided. A second related issue was the high number of weekly non-respondents that required to be called back – approximately 30% of the 700 clients at the HIV clinic and nearly 80% of the roughly 1600 ANC clients did not regularly respond to the weekly SMS message (some had already delivered). Calling back each client that did not respond to the follow-up weekly check-in text required a larger than expected budget for airtime, and the protocol was changed (similar results were reported from the Canadian projects). Only those with self-identified problems were then called back each week. A major challenge was phone connectivity and the inability of the offline system to organize and manage communications. Patient phones were frequently off due to lack of battery or being out of the service area. Tracking which numbers had been successfully contacted could not be done on the tablets, and required paper notes. Clinic staff emphasized the need to increase functionality of the tablet-based system to better manage patient communication, as had been developed in Canada.

All of this related to the perpetual need for tailoring and customization, for problem solving and co-development. However, there was a simultaneous recognition of the need to balance evidence-based changes with operational feasibility and streamlined functionality. Trade-offs needed to be considered in each adaptation (whether programmatic or platform-related), especially those that moved away from the initial “simplicity” of the RCT Kenyan trial. The push for further customization of the platform was highlighted in nearly all interviews. As one Canadian researcher put it, *“We had a lot of input into the new software. But already we want to add new features!”* This ranged from customized messages, greater functionality to manage the platform, inclusion of other health conditions and, critically, the ability to communicate test results by text. While some requests were unrealistic, or would play little role in changing health outcomes, others were actively taken-up in an instance of “co-design” with end-users, coupling research, clinic input and software development. One of the best examples included implementation in the ANC in Kenya, where Isiolo hospital managers wanted to include more versatile messaging:

*“We started with the Mambo program but then we switched…we wanted messages for all antenatal meetings. But [WelTel] said this was too hard. So we found a middle line.”*

Changes included reminder texts during different times of pregnancy, check-ins during the delivery period and then childhood immunization reminders.

One last area, important to scaling, related to small “glitches” that required IT support. Although WelTel V2.0 had run continuously for over a year at time of research, periodic issues did arise. Texts could sometimes “hang” for days in the network, influenced by the speed of the phone company, while messages could occasionally disappear and phone numbers get scrambled.

### Legitimization: The role of evidence and data

As we explored the ways in which WelTel projects generated funding and enrolled partners and collaborators, it became clear that data and evidence facilitated legitimacy to network across different global and local scales, and that this was key to scale-up. In effect, data and metrics functioned as a form of “organizational capital.”

Interviews stressed that the research data behind WelTel was “robust” and “some of the best out there.” The significance of the initial Kenyan RCT trial (published as editors choice in the *Lancet* ; cited more than 600 times) was often referenced. The WelTel Kenya1 RCT remains one of the only trials to show an improvement in biological markers for HIV using SMS, and it continues to receive positive reference [8]. This research focus led to obtaining multiple research grants, approximately $3 million stretched over 10 years, to adapt and evaluate WelTel in a range of contexts. A strong research track record of over 20 high-quality, peer-reviewed academic papers (proof-of-concept, health behavior, cost-effectiveness analysis, and RCT design) provided support.

Academic research helped obtain media attention, in Scientific American and CBC’s The National. It also elicited comment from the National Institutes of Health (NIH) Director, Dr. Francis Collins, during an mHealth Summit:

*“Clinical trials on mHealth ought to be the best way to determine what is actually working and what isn’t…there is no substitute…WelTel demonstrated an enormously important effect.”*

As a new field subject to crowding and competition, the emphasis on being “evidence-based” was seen as a way to distinguish WelTel from other mHealth applications. As one key WelTel staff member stated:

*“In the early 2000s, SMS was seen as a ‘no-brainer’ area to get into; so everyone got into it. Now it is a chaotic environment. Everyone is doing their own thing. But everyone is still in the pilot phase! Everyone. It is like all these different groups are creating their own word processors! …But people tend to overthink things. They want to have so many features, or have lots of information sent to the patient…[but] much of this stuff isn’t evidence-based…”*

WelTel could be “evidence-based” because it was amenable to clearly defined metrics: recruitment and adherence rates to therapies and appointments, text and phone records of patient-client interaction, clinic/hospital utilization and biological markers, such as CD4 count and viral loads for HIV. All current projects generated data aimed at this sort of evaluation.

However, moving from the controlled setting of a well-funded research study, often overseen by academic and NGO-staff, into the public health infrastructure in Kenya generated uncertainties with data and evidence that required negotiation. We found that quality of data, capacity for analysis and challenges in attribution were raised, with implications for data interpretation. For instance, a major issue was how to interpret data on “defaulters” in Kenya (someone who stops taking their HIV medication), which is important for evaluating the HIV clinic data. There are multiple reasons someone would default, or be counted as a defaulter. One major uncertainty related to what was believed to be a high number of patients that regularly switched clinics to avoid being seen or noticed. Defaulting rates some quarters were as high as 70% pre-WelTel, and there were clear difficulties with using the existing hospital-based data system for analysis due to errors and gaps in data.

Data and evidence, however, did not only play an important role at the global and national level as a form of organizational capital; they were also instrumental in generating legitimacy and buy-in from local clinic staff and management, albeit in a different way. A number of management staff in Kenya expressed that they were initially ambivalent or skeptical of WelTel until “seeing the data” from the first few months of activity at IDH:

*“At first, some thought this was just another NGO coming to benefit itself… but then we did a comparative analysis of fourth visits to the ANC after a few months and saw a big benefit…I took this data and did a presentation at county-level to convince them. Everyone was impressed, especially management level.”*

The example of the antenatal clinic data at Isiolo Hospital revealed how new forms of health data can play important political roles, which is well understood by local managers and staff. Improving MNCH indicators in Kenya is a major policy prerogative of the government and donors, especially in the NALs region. In 2013, the president put into place a new policy (The Beyond Zero Campaign) for free maternal care. Funding to the counties is based on a reimbursement system, and payments from central government are often delayed for months. If the hospital could keep better track of pregnant mothers and their delivery dates, so the reasoning went, funding could be more readily obtained from the central level. This was widely noted as one of the major motivating factors for IDH to push changes to the WelTel texting system in the antenatal clinic.

Interestingly, access to data also played a de-stabilizing role, whereby uncertainties of data security policies and laws generated barriers to scaling and integration in clinic routines. Patient confidentiality issues had informed the design of the WelTel service; phone numbers were not traceable to the clinic nor was HIV mentioned during the weekly routine texts (this was noted as very important for safety and mental wellbeing). For the Canadian projects, confidentiality was considered a *“Mount Everest issue”* that was hard to understand within the shifting legal regulations overseen by the health authority (Interview, Researcher, Canada). While it was easy to implement the WelTel intervention in a research context due to the clear protocols for informed consent, this was much less clear in routine care – a barrier that had prevented Oak Tree from fully implementing the service beyond ex-research participants when their research funding ran out.

### Scaling-up: Networks and policy contexts

Concerns about evidence and data were not the only factors shaping policy development – broader social, political and institutional drivers were considered equally, if not more, important. In our interviews, it was clear that successful scale-up would have to include the forming of new networks and linkages and the testing of different financing arrangements. The challenges involved are what make successfully breaking-out of the pilot stage so difficult in the mHealth field.

A bit of institutional history is informative. Partnerships were clearly an important mobilizing force for WelTel, grounded in a unique linkage of academic research, software development, clinical medicine and service delivery. This appears to be a common aspect of new mHealth platforms, especially transnational collaboration that spans both the Global North and Global South. The original RCT trial emerged from an international Manitoba-Nairobi network of HIV researchers, and took more than 3 years to finish. It also provided a different form of legitimacy, or “social capital”: sufficient depth of experience working in public health in Africa. As one Kenyan informant commented:

*“He [WelTel’s founder] has legitimacy. He has lived in Kenya, conducted clinical research in Kenya. He knows the country. He has friends. He is not seen as someone running in and running out just to benefit himself.”*

This experience facilitated strong networking capabilities, which explained the institutional history of WelTel. Moving from Kenya to the University of British Columbia (UBC) in Canada and the BC Centers for Disease Control (BC-CDC), WelTel’s founder was able to leverage the emerging interest in mHealth from Canadian researchers and funders. The Canadian projects were described as a type of “reverse innovation”, moving the Kenyan experience to vulnerable populations in Canada.

This also facilitated networking at the global stage, including participation on a number of advisory committees and contributions to international guidelines for HIV, TB and mHealth. This budding organizational visibility provided some unexpected opportunities – for instance, an Ethiopian postdoctoral student in Germany obtaining pilot financing from the Ethiopian Ministry of Health to trial WelTel in Gondar, Ethiopia. Other emerging opportunities included collaborations in South Africa, Zimbabwe, Uganda, Rwanda and the United States.

Implementing partners provided the necessary skills to facilitate service delivery and navigate local contextual terrains. One of the most significant collaboration involved links with Amref Health Africa, part of a large health consortium in Kenya (APHIA plus Imarisha) funded by USAID. Amref was described as a “legitimizing partner”, since the NGO has high visibility in Kenya and especially in NALs (where it has worked on MNCH and HIV). Amref was key in facilitating access to government leaders and clinics, and played a major role in obtaining the Grand Challenges Canada financing, providing significant in-kind contributions. Developing this collaboration took time, even after the grant started, as did setting-up human resources and partner networks in Isiolo county.

The benefits of partnering with established organizations were also noted in the Canadian context. Both of the Canadian HIV-projects, at Cedar and Oak Tree, were well-recognized organizations with established research experience, and with previous Canadian Institutes of Health Research (CIHR) grants. This had been important in their negotiation with a national telecom company to enable an affordable phone plan for vulnerable HIV patients, allowing unlimited text messaging and some call time without the typical conditions and terms.

However, the overarching challenge for WelTel and their partners was: how to move beyond the “research” stage? What did this mean, and how could it be done? Interviewees stressed the importance of forming new networks of partners especially with the business community and with focal Ministry of Health staff as a key pathway forward. But many of the key stakeholders, part of the WelTel partner network, tended to balance multiple responsibilities and had limited time for sustained engagement. One Canadian clinician-researcher highlighted the challenge of wearing multiple hats (balancing clinical responsibilities, research studies, searching for future funding, and networking with provincial authorities) while trying to bring the WelTel program into mainstream clinical care:

“ *I am a clinician, I have 30% time for research. I simply don’t have the time to chase up the heads of hospitals [to negotiate scale-up].”*

Others stressed the importance of having a full-time business director in order to seek out opportunities for growth in the private sector. Without core funding, this was difficult to arrange, and highlighted the different institutional capacity needs of a clinical-research network and a software company – as an organization, WelTel was, in effect, at a stage of development where it was balancing both.

The organization was described, after all, as a “social business.” Discussions centered on different financing arrangements, and how health impact in Africa could be approached sustainably while maintaining organizational growth. An ideal scenario, discussed by some, would be for clinics, hospitals and district and national health authorities to pay for the service, or at least co-finance it. In this scenario, adoption would be promoted based on an *intrinsic* value, which did not require augmenting staff salaries or supportive infrastructure (as had been done at Isiolo District Hospital). The incentives for health providers would be to help streamline work routines, avoid crises with patients, to receive feedback and recognition, and assist with work satisfaction. As one researcher mentioned in reference to Kenya:

*“WelTel can’t solve the human resource problem in Africa. The service helps with efficiency; it builds and reinforces existing capacity. It is not for us to plug huge gaps in capacity…Ideally, we want the service to be integrated into the budget lines of local health authorities.”*

However in order to achieve “buy-in” from local health authorities, cost data was needed.

*“The health system is not going to throw money at you! You need to prove that [WelTel] works. You need to use cost analysis to make the case for it and your health [care provider] utilization data has to show that it’s not too expensive. It’s about showing a “bang for your buck”…really, it’s about budgets.”*

(Interview, Clinician, Canada)

Cost data was repeatedly cited as the most important factor by county and hospital management in Isiolo, and by the Canadian teams, as fundamental to any discussions about sustainability. Maintenance costs were estimated to be very low – airtime for the HIV and ANC clinics, at Isiolo District Hospital in Kenya, were estimated at only $10 and $20 per month respectively, and roughly $300/month for the weekly SMS messages. In the Canadian HIV projects, discussions focused on the most vulnerable patients, who take up a disproportionate amount of inpatient time and resources. One researcher estimated that:

*“The most vulnerable [HIV-positive] patients [in Canada] will take up 30 or 40% of our time and resources but they are about 10% or so of our clients…so addressing prevention issues in this population with WelTel should have major cost-savings.”*

A cost analysis done by WelTel, which has been presented at major academic conferences, showed substantial savings (in the millions of dollars) for ART donors in Africa, extrapolated from the original Kenyan RCT findings. The asthma group at UBC in Canada noted how the disease affected over 3 million Canadians (with 500,000 having chronic obstructive pulmonary disorder) and cost the Canadian health system nearly $9 billion per year. Hence, researchers were quick to discuss scalability in terms of potential savings to the health system if low adherence rates could be addressed. However, interviews also revealed that this was an area that was still under-developed, and required more concerted work by health economists and business leaders to show cost-savings from mHealth interventions.

Aside from the need for cost data, there were different views about the willingness and ability for hospitals and clinics, in both Africa and Canada, to finance WelTel directly. The CDC demonstration study in Kenya had aimed to have the system sustained and financed by eight hospitals in Nairobi. However, in practice, nurses had to be financially compensated for their extra time, and the clinic management (although very supportive of the service) did not commit the necessary resources.

*“These places did not want to provide airtime. That was it…the evidence was there; they loved us. But you find a major disconnect between the clinic staff who saw the day-to-day benefits and the managers that directed funds…We were pitching ideas but they weren’t taking them.”* (Interview, Kenya)

Certainly, there were challenges in engaging management of African health systems to support health technology innovation when basic infrastructure and staff salaries were difficult to maintain. In Isiolo, user fees could have been used to budget for the (modest) cost of airtime to support WelTel. However, with the devolution of local government in the new 2010 constitution all funds were decentralization and managed by the county office. To better address these issues, a “WelTel sustainability committee” had been organized in Isiolo in 2015. Staff and airtime from the county budget were tentatively promised, but major political norms and a lack of technical capacity problematized their realization. As one interviewee discussed:

*“There are many problems with this idea [of having the county pay for WelTel services]. The major one is a perception problem: politicians like roads – things you can see…There is already a perception in county government that health receives too much money…[and secondly], county officials do not prioritize technology…There is a gap in appreciation of technology.”*

Discussions at the county level in Isiolo revealed that health sector funding is taken-up overwhelmingly with staff salaries, with the limited field budgets directed to per diems, fuel and drug costs. Government officials noted that they were most interested in technology management platforms that would allow them to track leakages in procurement, revenue collection and “ghost workers.” Going back to the example of IDH, decision-makers were most interested in how technology could improve existing systems and not necessarily focused on patient-centric approaches. Additionally, there was a pervasive perception, set by historic-political precedence, for commodities (i.e. drugs) and other supportive innovations to come from central government or donors. Donors, such as PEPFAR, provide most HIV drugs in Kenya, and other clinical services, like psychosocial support, are seldom paid for by clinics and hospitals, especially in poorer regions like NALs. Rather, they are provided through grants to NGOs like Amref, which are intermittent and dependent on shifting donor priorities. Generating major donor or government support would require, as one informant stated, “high-level commitments and a system ready to deploy massively [at scale]” (Interview, Isiolo).

Hence discussions around financing led to concerns about the need to “package” the WelTel service for policy uptake. Ambitions were aimed at scaling WelTel across Africa as part of HIV, TB and ANC services, and in some other form of commercialization for the North American market. Various business consultants had been brought in, often on a *gratis* basis, to explore this. A free licensing model for resource-limited settings supported by technical support contracts had been adopted, which would model Socially Responsible Licensing (SRL) principles. However as a new field, mHealth applications and supportive policies were noted to be still nascent and fragmented, and willing-to-pay rather uncertain. As a WelTel-associated researcher discussed:

*“People expect mHealth to be based on tiny budgets, or that it should all be provided for free, open source. Everyone wants to do their own thing. But ARVs are not open source! It’s an innovation that needs to be provided, like a business…like a social business.”*

The WelTel software was provided for a licensing fee to each of the individual research projects, but some clinicians stressed that this expense would be hard to justify year-after-year at the facility-level:

*“It would be better if it was something like Microsoft Word where you buy it and you can just use it…you can even modify it with upgrades as you go along.”*

(Interview, Clinician, Canada)

Interviewees also highlighted some challenges in “selling” the service to policymakers. In Canada, perhaps one of the most challenging questions was related to the ability to convince policymakers that vulnerable HIV-positive clients should be given a cell-phone (which had been the case with the two HIV projects):

*“You can clearly see the impact [of the WelTel intervention and phone provision] but how do you convince people to give money to give a drug user a phone? It is a hard sell!”*

Some believed that engaging in auxiliary technology features (such as the provision of phones) could distract from the aims of rapid scalability. To others, however, it was essential to target the most vulnerable patients. Hence, there was still a sense that the organization was wresting with the proverbial question: *what type of service should WelTel be offering and to whom?* There was also an ongoing debate about how to link public health impact with business interests and organizational growth.

## Discussion

While the mobile phone revolution has enormous potential for public health, both in terms of improving patient adherence to therapy and outcomes, and wider health system strengthening, current evidence of successful scale-up and impact is still in its infancy ([9, 11, 28]). The promise of investing in mHealth may offer a unique opportunity in developing countries due to the minimal infrastructure requirements comparable to e-health [29] but for this to be realized, systemic health system weaknesses need to be understood, navigated and negotiated. Furthermore, there needs to be a deliberate effort by funders and governments, in both the Global South and Global North, to combine the enthusiasm for scaling with the rigors of evidence-based practice – less scaling is viewed more as market growth by industry than as an opportunity to benefit public health [12]. This makes understanding real-world programmatic challenges, and the individual institutional histories of particular mHealth innovations – how networks form, evolve and change – all the more urgent.

In a recent systematic review of mHealth in Africa, Chib et al. [9] distilled lessons learned from 44 projects, most all of which were small-scale. They found that appropriate project design, stakeholder participation, integration with the health care system and the subsequent use of appropriate technology and resources were key factors in successful implementation. In contrast, they noted how lack of funds, unknown cost-effectiveness and lack of evidence inhibit scalability. A second review, Gagnon et al. [30] analyzed 33 mHealth studies on individual, organizational and contextual factors influencing healthcare provider adoption. They highlighted the importance of usefulness, ease of use, design, cost, time, and a variety of related factors.

In this paper, we have provided a nuanced and contextualized perspective on these issues, using the Consolidated Framework for Implementation Research (CFIR) as a guiding conceptual lens to explore the implementation process for WelTel in both resource-limited (Kenya) and resource-rich (Canada) settings. Through this qualitative and comparative approach, we have discussed various aspects of the complexity of implementation and how they are linked at multiple levels. As the WelTel service and other initiatives move forward, implementation of mHealth, as a disruptive and positive change technology could benefit substantially from further in-depth case study evaluations.

However, the benefits of unpacking the textured social factors integral to moving mHealth innovations forward should not end with knowledge generation and academic publishing. Closing the “implementation gap” in public health [31] requires coupling research on operational issues with pathways for “actionable intelligence” – feedback loops that can, in the maze of factors that can derail or enhance an implementation pathway and organizational network, help guide stakeholders towards new ways of problem solving.

As with many global health innovations, one important challenge for the mHealth community going forward, including for WelTel, is to maintain flexibility and adaptability in the technology platform and service delivery pathway when moving from proof-of-concept to wider scale-up [31]. This is the classic transplantation challenge in public health – when scale-up occurs, the careful attention to detail that facilitated the initial success becomes marginalized [25]. The organizational culture of the project is challenged with the complexities of growth; for example, each implementing clinic will still require a local champion to ensure effective implementation. Hence, harnessing mHealth to impact population-based health outcomes will require thinking carefully about how interventions interact with, and influence, their social milieu. As we have shown in this paper, this includes diverse stakeholder interests, clinic work routines, local organizational cultures and broader health systems context.

## Conclusion

Realizing the health benefits of the cell phone revolution remains an area for concerted work. The danger is that an over-emphasis on technological innovation, on the “bells and whistles”, as Tomlinson et al. [12] called them, can obscure the coupling of technical, social and political dynamics essential to effective implementation and long-term scale-up. The drive towards “the next best thing” could mean that funders and innovators abandon mHealth platforms, or do not provide sufficient pathways to growth, just as they are beginning to show promise in terms of scalability. Even now, some view mHealth as “old-news”, while others remain skeptical or unsure of how, and in what ways, mHealth platforms can effectively move into the commercial market, especially for vulnerable populations, whether in Africa or North America. This is all to say that research networks that have developed still nascent but promising products, like WelTel, are placed in a tricky position. As we have endeavored to show in this paper, constant innovation is required not only in technology but also in network building, institutional growth and partnership development.

## Footnotes

1 Where quotes are not qualified by participant category, it is because the statement was used by multiple participants and represents a widely shared perspective.
